# War on Disease

**Published:** 1920-10-16

**Authors:** 


### War on Disease.

### NATIONAL AND INTERNATIONAL CO-OPERATION.

The national appeal which is being made in the second week of October by the British Red Cross Society and kindred societies in support of every phase of health work from infant welfare to wounded Service men, provides an opportunity to take a wider view of Red Cross activities as represented by the League of Red Cross Societies, to which the British Red Cross Society is affiliated. In this way it is possible to emphasise the great scope of the work and the national and international importance of each country's branch of the League playing its proper part in its own sphere. We have before us the most recent number of the *International Journal of Public Health*—a most valuable publication—which, among other things, contains a record of the first meeting of the Medical Advisory Board of the League. It covers much ground. Diseases ravaging Poland, the deplorable sanitary conditions of Eastern Europe, the publication of scientific results, the collection of public health data, relief work, propaganda and education, child welfare, tuberculosis, nursing sanitation, research, statistics, venereal disease, malaria, etc., are among the subjects on which this international organisation

**THE PUBLIC WELL-BEING**—(*continued*).

is engaged, and though some of the work will eventually fall within the scope of the health organisation to be set up by the League of Nations, there is a great range of labour for this central organisation of national societies in supplementing and assisting the official international health organisation. We append a summary of two or three of the resolutions, so that some idea may be gathered of their value and significance, and of the trend of policy.

### Child Welfare.

Puericulture (child welfare) consists not only in the care of sick children, but more especially in the measures as a whole to be adopted with regard to children *before and after birth*, in order to ensure normal development and avoid illness. With this object child welfare must, in the first place, be based on the special training of doctors and the education of the public.

National Red Cross Societies should take measures to develop among nurses training in puericulture. The League should, in conformity with its resources, participate in this activity, either by creating or assisting in the creation of schools of puericulture, or child-welfare centres, or by providing scholarships for doctors and nurses at existing schools of puericulture, such as the Franco-American School of the Faculty of Medicine in Paris or similar schools in other countries, and, finally, by the dissemination of the means of educative propaganda among the people.

It was resolved that a child-welfare unit, of the nature recommended by the Child Welfare Department of the General Medical Department of the League of Red Cross Societies, be immediately organised and sent into that country whose national Red Cross Society would engage gradually to take over the work inaugurated by this unit, and eventually to duplicate its personnel, activities, and material.

The Medical Advisory Board consider it desirable to support a limited investigation into the causation of rickets during the present opportunities provided at Vienna, as indicated in outline by Sir Walter Fletcher on behalf of the British Medical Research Council.

### Tuberculosis.

So far as concerns the participation of the League in the world campaign against tuberculosis, the Medical Advisory Board consider that the League should proceed to create, in certain districts of one or several of the countries of Europe that are most seriously affected with tuberculosis, an experimental organisation on a more or less limited basis, expressing in its constitution a part of the general programme. This part should include :

1. A preliminary survey of latent tuberculosis as well as of open infection in the entire population of some chosen districts, according to age, sex, profession and social groups;

2. A statistical study of tuberculosis mortality and of the various forms and localisations of the disease (in co-operation with the local public health authority);

3. The establishment of an adequate number of dispensaries managed by specially trained physicians and visiting nurses;

4. The organisation of sanatoria for the isolation of advanced cases and for treatment of curable cases;

5. The creation of preventoria and open-air schools for children;

6. The education of the medical profession as well as of the general public regarding the social fight against tuberculosis.

It was resolved that the League of Red Cross Societies organise an anti-tuberculosis demonstration in one or several countries where this demonstration would be particularly desirable and where the national Red Cross Society would bind itself to continue and to develop the movement undertaken.

### Sanitation.

That, owing to the importance of a clean, sanitary environment in maintaining health conditions and in preventing the spread of communicable diseases, the general programme of sanitary surveys and other activities submitted by the Department of Sanitation of the General Medical Department of the League of Red Cross Societies be approved.

That the national Red Cross Societies exert their influence to obtain, where such needs arise, hygienic habitations, pure water supplies, safe methods for the disposal of human excreta and other refuse, together with various other sanitary improvements.

That each national Red Cross Society have in its organisation one or more competent persons who will devote themselves specially to this branch of the work.

That sanitary surveys be encouraged and undertaken for the purpose of obtaining information in regard to all matters pertaining to public health, creating a general interest in sanitation, and serving as a basis for undertaking necessary sanitary improvement.

That demonstrations of the efficacy of water purification by chlorination, as a means of preventing water-borne diseases, be made under the auspices of the League of Red Cross Societies in co-operation with the authorities at strategic places, especially in cities which are ports and railroad centres.

### Venereal Diseases.

The Medical Advisory Board consider that it would be desirable for the League of Red Cross Societies to devote its attention to the question of education in sex hygiene and anti-venereal propaganda. The Board consider besides that it would be suitable for the League to study the means by which it would be possible to reduce the cost of specifically anti-syphilitic drugs which bring about the prompt disappearance of contagious lesions.

The Board consider that the diffusion of moral and physiological knowledge and of the ideals of purity and of the integrity of family life cannot be too much encouraged. It was resolved :

That whereas venereal diseases are prevalent and dangerous communicable infections against which science has developed a practical programme for eradication, the League of Red Cross Societies (*a*) recommend to all national Red Cross Societies the desirability of holding annually, or at frequent intervals, regional conferences upon this subject for friendly review and criticism of the measures proposed, and (*b*) tender its services to all countries desiring to participate in the organisation of such regional conferences.

It is obvious that if the League is to have its full effect the national societies must be supported to the fullest possible extent.

